# Adjusting Assistance Commensurates with Patient Effort During Robot-Assisted Upper Limb Training for a Patient with Spasticity After Cervical Spinal Cord Injury: A Case Report

**DOI:** 10.3390/medicina55080404

**Published:** 2019-07-24

**Authors:** Kenichi Yoshikawa, Kazunori Koseki, Yusuke Endo, Satoshi Yamamoto, Kyoko Kanae, Ryoko Takeuchi, Arito Yozu, Hirotaka Mutsuzaki

**Affiliations:** 1 Department of Physical Therapy, Ibaraki Prefectural University of Health Sciences Hospital, 4733 Ami, Inashiki-gun, Ibaraki 300-0331, Japan; 2 Department of Physical Therapy, Faculty of Health Science, Health Science University, 7187 Kodachi, Fujikawaguchiko-machi, Minamitsuru-gun, Yamanashi 401-0380, Japan; 3 Department of Physical Therapy, Ibaraki Prefectural University of Health Sciences, 4669-2 Ami, Inashiki-gun, Ibaraki 300-0394, Japan; 4 Department of Orthopaedic Surgery, Ibaraki Prefectural University of Health Sciences Hospital, 4733 Ami, Inashiki-gun, Ibaraki 300-0331, Japan; 5 Department of Rehabilitation, Ibaraki Prefectural University of Health Sciences Hospital, 4733 Ami, Inashiki-gun, Ibaraki 300-0331, Japan; 6 Center for Medical Sciences, Ibaraki Prefectural University of Health Sciences, 4669-2 Ami, Inashiki-gun, Ibaraki 300-0394, Japan

**Keywords:** spinal cord injury, robot-assisted training, upper limb, effort, optimal assist, spasticity

## Abstract

Limited evidence is available on optimal patient effort and degree of assistance to achieve preferable changes during robot-assisted training (RAT) for spinal cord injury (SCI) patients with spasticity. To investigate the relationship between patient effort and robotic assistance, we performed training using an electromyography-based robotic assistance device (HAL-SJ) in an SCI patient at multiple settings adjusted to patient effort. In this exploratory study, we report immediate change in muscle contraction patterns, patient effort, and spasticity in a 64-year-old man, diagnosed with cervical SCI and with American Spinal Injury Association Impairment Scale C level and C4 neurological level, who underwent RAT using HAL-SJ from post-injury day 403. Three patient effort conditions (comfortable, somewhat hard, and no-effort) by adjusting HAL-SJ’s assists were set for each training session. Degree of effort during flexion and extension exercise was assessed by visual analog scale, muscle contraction pattern by electromyography, modified Ashworth scale, and maximum elbow extension and flexion torques, immediately before and after each training session, without HAL-SJ. The amount of effort during training with the HAL-SJ at each session was evaluated. The degree of effort during training can be set to three effort conditions as we intended by adjusting HAL-SJ. In sessions other than the no-effort setting, spasticity improved, and the level of effort was reduced immediately after training. Spasticity did not decrease in the training session using HAL-SJ with the no-effort setting, but co-contraction further increased during extension after training. Extension torque was unchanged in all sessions, and flexion torque decreased in all sessions. When performing upper-limb training with HAL-SJ in this SCI patient, the level of assistance with some effort may reduce spasticity and too strong assistance may increase co-contraction. Sometimes, a patient’s effort may be seemingly unmeasurable; hence, the degree of patient effort should be further measured.

## 1. Introduction

Spinal cord injuries (SCIs) are caused by accidents and violence worldwide [1], and the annual incidence rate in Tokushima Prefecture in Japan is 40.2/100,000 [2], and that in the United States is about 12,000 new patients [3]. Spasticity is one of the major secondary complications of SCI patients, and spasticity is a phenomenon that occurs as resistance to passive, velocity-dependent resistance of a muscle to stretching [4]. The cause of spasticity is multifactorial and results from the impairment of complex nerve actions [4]. In addition, it may be suggested to be associated with co-contraction of the arm during extension movement, and a report suggested that such a phenomenon may be a hindrance to daily movement [4,5]. For improvement of spasticity, pharmacological prescriptions are usually made, and voluntary movement training in physical therapy may also produce good effects [4].

The development of the research on robot-assisted training (RAT) for the treatment of spinal cord injury (SCI) is progressing rapidly in clinical situations requiring voluntary movement training. Robot control strategies for central nervous system (CNS), including SCI, recovery are often applied in motor learning. In principle, for neuroplasticity and motor learning, robot assistance for training needs to be variable, and patients need to experience actual tasks as much as possible [6,7]. In other words, robot assistance may need to be minimized to the extent that patients do not reduce the opportunity to exert effort. Thus, studies focusing on the generation of assist-as-needed robotic therapy are necessary [8,9]. Although effort and volitionality are important elements in neuroplasticity [10], only a few studies have reported on how much effort a patient has actually exerted during RAT, so the optimal patient effort and degree of assistance to attain good changes for neuroplasticity, including improvement of spasticity, in the patient’s condition remain unknown. The same clinical question can be applied to the upper limb single-joint hybrid assistive limb device (HAL-SJ; HAL-FS01, Cyberdyne, Inc., Tsukuba, Japan). HAL-SJ has a bio-electrical signal (BES)-based control system and demonstrates joint torque assist with the wearer’s voluntary drive. With regard to training using HAL-SJ, to the best of our knowledge, there has been no report on assist adjustment based on the degree of patient effort.

Given the limited evidence on this theme, this case report showing preliminary feasibility aimed to investigate the optimal relationship between the extent of patient effort and robotic support. We performed training using an electromyography (EMG)-based robotic assistance device in one patient with cervical SCI under multiple conditions adjusted to patient effort. In this patient with SCI, we showed immediate changes in muscle contraction patterns, patient effort, and spasticity.

## 2. Case Presentation

This study was carried out in accordance with the Declaration of Helsinki, with approval from the Ethics Committee of the Ibaraki Prefectural University of Health Sciences (approval number: 797; date of approval: 28 December 2017). Written informed consent was obtained from the patient for publication and the use of accompanying images in this case report.

A 64-year-old man (height: 162 cm; weight: 59.1 kg) was diagnosed with cervical SCI after falling from a horse. At the time of injury, the neurological level of injury was the fourth cervical (C4) level, and the manual muscle test (MMT) score was less than 1 in areas beyond the injury level. Third to sixth cervical laminoplasty and seventh cervical laminectomy were performed on day 26 after injury. At post-injury day 32, lower limb muscle strength recovered to MMT grade 2 or 3. He was transferred to another hospital to continue rehabilitation. He then received physical therapy and occupational therapy on post-injury day 59. However, there was no significant change in muscular strength. He was then transferred to our hospital on post-injury day 206 for further rehabilitation, and his house was renovated in preparation for community care services upon discharge. On evaluations of physical function at the time of admission to our hospital, using the International Standards for Neurological Classification of SCI [11], the American Spinal Injury Association Impairment Scale was C, neurological level was C4, and motor scores at right and left upper limb key muscles (from C5 to T1) were 4/4 (right/left), 4/3, 3/3, 2/2, and 3/3, and those at lower limb key muscles (from L2 to S1) were 3/3, 3/3, 3/3, 3/0, and 5/3, respectively. His tactile sensory assessment by light touches was normal until C6, impaired from C7 on both upper limbs, and absent on the entire lower limbs. Pain sensation by pin-prick was normal until C4 at the right side and until C6 at the left side, but absent on the caudal side from these levels. Spasticity measured by modified Ashworth scale (MAS) was 2 and 1+ at right and left elbow flexors, respectively, and 1+ and 1 at wrist flexors, 1 and 1 at knee flexors, 1+ and 1+ at knee extensions, and 0 and 1 at ankle dorsiflexion upon admission to our hospital. There was no limitation of the passive range of motion of the elbow joint. Spasticity and co-contraction of his biceps brachialis muscle often made elbow extension difficult, which fluctuated daily. There were no medication changes affecting relief of spasticity during the evaluation for this report and during HAL-SJ training.

### 2.1. Intervention

**Figure 1:**
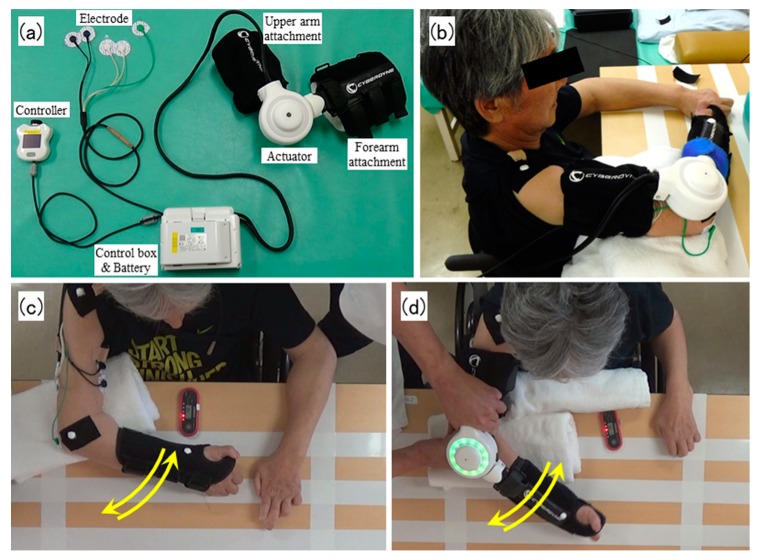
Single-joint hybrid assistive limb device (HAL-SJ) overview and the task with and without HAL-SJ. (**a**) An overview of HAL-SJ. (**b**) Side view of training using HAL-SJ. Images (**c**) without HAL-SJ and (**d**) with HAL-SJ show the horizontal motion of the elbow seen from above. HAL-SJ, upper limb single-joint hybrid assistive limb.

Using HAL-SJ, it is possible for the wearer to voluntarily perform active assistive exercises by BES-based control (Figure 1a). Normally, the BES of antagonist and agonist muscles is used for controlling HAL-SJ. Using the hand controller, the sensitivity adjustment of the amount of assist torque according to BES can be increased or decreased with the control item “assist gain.” Assist gain can be set every 10 from 0 to 100, to assist joint movement and controlled by the therapist by operating the handy controller. While confirming the degree of effort of the wearer, assist gain can be adjusted to set a desired assist. However, with CNS disorders, the normal muscle output may not be expected because of pathological muscle synergy or spasticity. If the assist motion based on BES is not successfully performed under abnormal BES due to abnormal muscle co-contraction resulting from CNS damage, the BES can be cut at 20 levels of 0% to 100% at every 5% to cancel out undesirable assistance due to the abnormal BES by the antagonist muscle when using HAL-SJ. This control item is called “assist balance,” which makes it possible to adjust the balance of the assist torque of flexion and extension by cutting the signal of the antagonist or agonist muscle. The assist balance can also be adjusted by the handy controller. For example, considering imbalance of muscle contraction or muscle tone of antagonist and agonist muscles, the proportion of the signal of the muscle producing stronger BES can be cut to balance out the resulting torque extension/flexion. As a result, the wearer can reproduce a motion close to normal. Details of the HAL system were previously presented [12,13].

**Figure 2:**
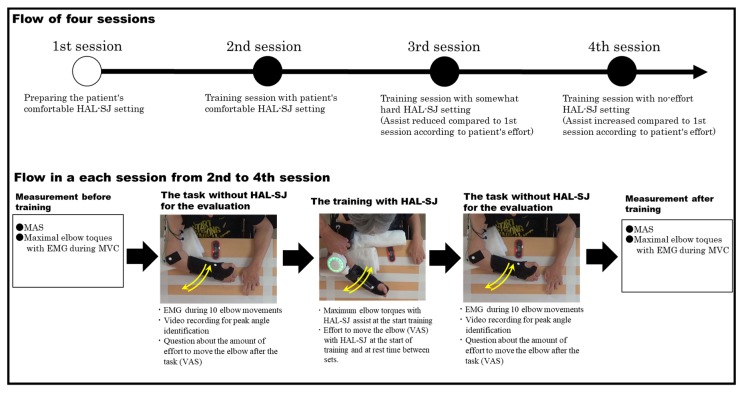
Flow of the whole study and procedures in each session.

Figure 2 shows the flow of the whole study (top part of the figure) and the procedures in each session (bottom part of the figure). The starting position of the training task was 90° shoulder flexion and 90° horizontal adduction, and 0° rotation on the table with 90° elbow flexion (Figure 1b). The upper arm was fixed to the table, HAL-SJ was attached to the elbow joint, and BES for assist control was derived from the biceps brachii and triceps brachii muscles. The BES sensor was placed on the muscle berry of each muscle so as not to touch the EMG sensor. The elbow was fully extended and flexed 90° on the horizontal surface to match the 30 beats per minute (bpm) rhythm of the electronic metronome sound (completing one extension and flexion once in 4 s) (Figure 1c,d). The patient was fixed to the back of the wheelchair. Until the end of hospitalization, RAT was conducted for a total of four sessions. The first and second sessions were held at post-injury days 403 and 405, respectively. We set up more than five days between the second and third (post-injury day 410) sessions and between the third and fourth (post-injury day 417) sessions.

The patient had no experience on robot-assisted training, including HAL-SJ, by this time. In the first session, we decided to set the HAL-SJ by adjusting “assist gain” and “assist balance” to allow the patient to perform elbow extension and flexion exercises most comfortably for five sets with 10 times per set. We defined comfortable assist settings as allowing the patient to move the elbow as he thought. During this task, we recorded the degree of effort of extension and flexion with the most comfortable HAL-SJ setting using the visual analog scale (VAS) (0, no difficulty at all; 10, completely fixed and not moving at all). In this session, the patient got used to the practice environment and the task method, and the VAS score was 1 when he moved his elbow with HAL-SJ. In the second session, HAL-SJ training was conducted with the most comfortable HAL-SJ setting. Considering the possibility that the BES for the HAL-SJ changes slightly depending on the physical condition and skin resistance, the HAL-SJ setting was fine-tuned to the same degree of effort (same VAS value 1) as in the first session. In the third session, the “assist gain” of the control item was decreased (assistance by HAL-SJ was reduced), and the degree of effort of the task was 2 on VAS. With this setting, the patient demonstrated that exercise was slightly difficult (VAS: 2). In the fourth session, the “assist gain” was high compared to that in the first session, and the assignment effort was VAS: 0. In the second, third, and fourth sessions, 10 times × 6 sets of tasks were performed. In the second and subsequent sessions, the extent of effort (VAS) to move the elbow with assistance using HAL-SJ during training was confirmed at the start of training and at rest time between sets. During the break between sets, the assist gain item was fine-tuned according to the person’s complaint, so that the level of effort (VAS score) during HAL-SJ training became constant.

### 2.2. Outcome Measure

In the evaluation, the same movement as the above-mentioned training task was carried out 10 times at 30 bpm without HAL-SJ just before, and immediately after, training at every session. EMG patterns of the biceps brachialis and triceps brachii muscles were recorded using a wireless surface electrode with a sampling rate of 2000 Hz and bandpass filtering of 20–450 Hz (Trigno Lab, Delsys, Inc., Boston, MA, USA). EMG patterns at maximum voluntary contraction (MVC) of the biceps brachialis muscle and triceps muscle of the upper arm for 5 s or longer were recorded. In addition, the duration of the task was video recorded from the top to the bottom of the patient’s upper limb and synchronized with EMG data for evaluation (60 Hz, HDR-CX 470, Sony Marketing, Inc., Tokyo, Japan). Changes in the movement of the elbow flexion angle were calculated from the motion picture obtained by the motion analysis software (Frame-DIAS V, DKH Co., Ltd. Tokyo, Japan), and peaks of angles of 10 elbow flexions and extensions were identified. All acquired EMG data were full-wave rectified and integrated every 300 ms (iEMG). To remove motion artifact, EMG data were filtered by a digital high-pass of 4th order Butterworth filter with a cut-off frequency of 0.5 Hz. EMG data were also filtered by a digital band-pass filter of 4th order Butterworth filter with a cut-off frequency of 20–450 Hz. Zero-phase forward and reverse digital filtering was achieved using Matlab routine program “filtfilt,” provided by Matlab Signal Processing Toolbox. Appropriate iEMG data for 2 s during MVC were identified and averaged for 2 s (100% MVC). The iEMG data during movement were divided by the 100% MVC value to calculate the %MVC data. The peak point of the bending and stretching angle was taken as the switching point between flexion and extension, and EMG data were divided into 10 paired extension and flexion phases. The time of each data was normalized to 100 points, and the data of 10 phases were averaged. EMG data were processed in MATLAB R2018a (MathWorks Inc., Natick, MA, USA). Furthermore, immediately before and after the training at the second to fourth sessions, the extent of effort required during elbow movement without HAL-SJ was recorded, and the spasticity of the right biceps brachii muscle and triceps brachii muscle was evaluated by MAS. The maximum isometric torque of elbow joint extension and flexion was evaluated using a handheld dynamometer (MicroFET2; Hoggan Scientific LLC, Salt Lake City, UT, USA) at the start of training with HAL-SJ and without HAL-SJ immediately before and after training. The assist rate was calculated by dividing the maximum torque with HAL-SJ by the maximum torque immediately before training without HAL-SJ. Once EMG electrodes were attached at the beginning of the session, they were not removed until all measurements in the session were completed. The placement of the EMG electrodes was the same throughout the session and was marked according to the SENIAM project [14]. EMG results were compared before and after training by graphical visual analysis of the change in the degree of contraction of the antagonist muscle and that of the agonist muscle. Values of MAS, VAS (degree of effort), and maximum elbow torques were compared immediately before and after each session. All assessments were performed by the same physical therapist.

## 3. Results

All sessions were safely completed without adverse events. The assist torque setting at the assist balance was 20% at the biceps and 100% at the triceps during all sessions and all training sets. Table 1 shows outcomes before and after HAL-SJ training. In the MAS, the spasticity of the right biceps decreased by one at the second and third sessions, and the spasticity of the right triceps did not change at all sessions. In the MAS score of the left hand, there was no change at the second and third sessions. The fourth session improved the MAS by one level, even though the right hand had no change before and after training. Comparison of results of before and after training elbow joint torque without HAL-SJ showed that the extension torque was almost unchanged, but the flexion torque decreased in all sessions. Elbow extension and flexion torques just before training with HAL-SJ were 311% and 98% in the second session, 249% and 105% in the third session, and 282% and 104% in the fourth session, respectively.

Table 2 shows the extent of effort (VAS) before and after training without HAL-SJ and during HAL-SJ training. VAS scores of the patient’s efforts during task training with HAL-SJ from the second to the fourth sessions were 1, 2, and 0, respectively.

**Figure 3:**
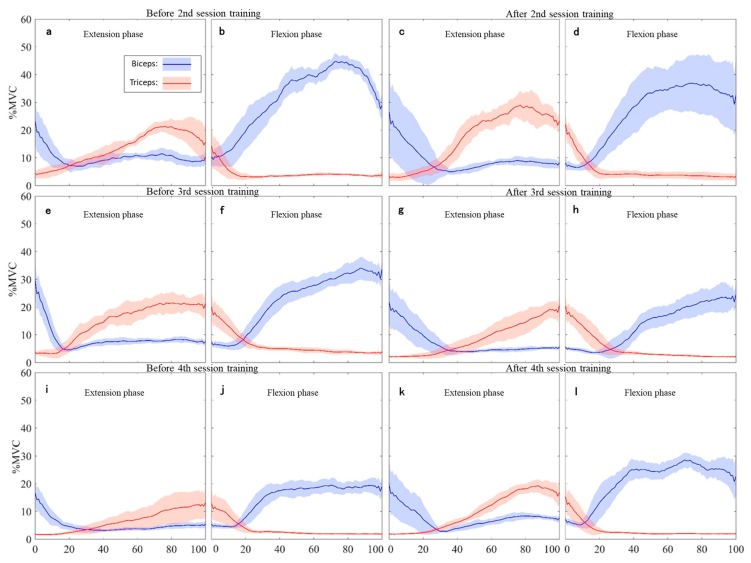
EMG (%MVC) of biceps and triceps muscle just before and after HAL-SJ training. These graphs show averaged %MVC of 10 motions (solid line) and standard division (light-color-filled area). Graphs (**a**) and (**b**) respectively show the extension phase and flexion phase before training, and (**c**) and (**d**) show similarly after training in the second session. Graphs (**e**) and (**f**) respectively show the extension phase and flexion phase before training, and (**g**) and (**h**) show similarly after training in the third session. Graphs (**i**) and (**j**) respectively show the extension phase and flexion phase before training, and (**k**) and (**l**) respectively show similarly after training in the fourth session. EMG, electromyography.

On the evaluation of co-contraction using EMG visual analysis, the co-contraction of the biceps brachialis muscle of the extension phase was markedly decreased before and after HAL-SJ training on the third session (Figure 3e,g). The co-contraction of the biceps brachialis during extension phase slightly decreased before and after HAL-SJ training in the second session (Figure 3a,c). However, the co-contraction of the biceps brachialis increased in the extension phase at the fourth session (Figure 3i,k). In the flexion phase, there was no difference in the co-contraction of the triceps brachialis muscle before and after training and between sessions (Figure 3b,d,f,h,j,l). Comparison results of the degree of contraction of the agonist muscle before and after the HAL-SJ training in the extension phase showed that %MVC of the biceps decreased only in the third session and increased in other sessions (Figure 3e,g). In the flexion phase, the %MVC of the triceps decreased in the second (Figure 3b,d) and third (Figure 3f,h) sessions, and increased in the fourth session (Figure 3j,l).

## 4. Discussion

The results of MAS and EMG showed a reduction in spasticity and co-contraction only in the second and third sessions of HAL-SJ training that require patient effort. In the extension phase before the third training session, EMG showed strong contraction of the upper arm biceps muscle from the start of contraction, with a VAS score of 9. Although these results suggest that elbow extension was difficult before training at the third session, the elbow movement without HAL-SJ after training became easier (VAS: 9 to 2), with improvement in the co-contraction of the antagonist muscle and spasticity. In the fourth session, the results of MAS and EMG showed no change in spasticity and enhanced the co-contraction of the antagonist muscle. In principle, in the rehabilitation of stroke patients with elements of treatment similar to SCI, effort and volitionality are important elements in neuroplasticity [10]. Furthermore, in stroke patients with spasticity, power and effort may be misidentified [15], and assistive exercise provides a new somatosensory stimulus that helps induce neuroplasticity when exercise with voluntary effort is difficult [16,17]. Shimizu et al. reported improvement in spasticity in cerebral palsy individuals on HAL-SJ training, and they explained that this improvement, in the viewpoint of motor learning, was attributed to HAL training, which allows near-normal movements, movement driven by muscle activation, focused movement, repetition of desired movements, and training specificity [13]. HAL-SJ training, where joint torque assistance in response to muscle activation is applied in real time, helps achieve these features with proper coordination [13]. Similarly, in this study, the second and third session settings required some extent of effort from the patient, and near normal movement was assisted according to the effort of the patient, which corresponded to the appropriate amount of muscle activation. These factors during training may have led to an immediate and timely improvement of spasticity and co-contraction. Co-contraction was enhanced after HAL-SJ training in the fourth session. Interpreting this result in terms of motor learning in the same manner as described above, an assist that is too strong may not have been able to learn normal joint movement control due to the absence of voluntary effort. Thus, for this patient, too strong HAL-SJ training was not a desirable exercise and might have enhanced co-contraction of antagonist muscles. To our knowledge, no study has shown how the degree of effort of patients with cervical SCI using HAL-SJ relates to spasticity and co-contraction.

Although the assist rate for elbow extension torque with HAL-SJ was expected to be the highest in the fourth session of excessive torque setting, the assist rate at the fourth session was interestingly less than that in the second session under a comfortable torque setting. The fact that the assist rate during the fourth session is lower than that during the second session means that the assist required for the patient to perform the task exercise has changed. The reduced assist required for self-elbow extension of patients experiencing spasticity and co-contraction of antagonist muscles also enhanced the aforementioned consideration that improvement in MAS and co-contraction was brought about by motor learning training at the second and third sessions. In the future, it will be necessary to investigate in detail whether such changes are associated with neurophysiological changes such as reciprocal innervation. In addition, this change in assist might be brought about by previous sessions due to the relearning of power and effort. However, the patient’s ease of voluntary extension, which was observed to have improved once, was not maintained with the fourth session’s too strong assist of HAL-SJ. With this result, focusing on the fact that it led to undesirable results despite the reduction in required assistance is important. From this point of view, we consider that clinicians should always observe the patient’s level of effort and adjust the level of assistance in response.

In the same way as above, factors that reduced the maximum flexion torque before and after training in all sessions may have caused excessive muscle output of the brachial biceps, which was corrected by relearning force and effort, and there was also the possibility of torque reduction by muscle fatigue.

With regard to the MAS score of the left hand, there was no change in MAS before and after training in the second and third sessions. The fourth session improved the MAS by one level, even though the right hand had no change before and after training. Shimizu et al. reported a case report of HAL-SJ training on two late-teen patients with cerebral palsy [13]. In one patient, HAL-SJ training was performed on the unilateral upper extremity. As a result, although there was no improvement in MAS on the non-interventional side of HAL-SJ, the non-interventional upper arm function (Action Research Arm Test score) was improved. However, because the report by Shimizu et al. differed from our report in age, disease, frequency of intervention, and assist setting, and it was a case report, it is not possible to identify the cause of the effect on the non-interventional side for now. In the future, it is necessary to elucidate the mechanism of the influence on the non-interventional side.

In general, using a robot with less effort can make it easier to increase the number and amount of exercise. Although it is important to contribute to neuroplasticity by increasing the frequency and performing the task repeatedly [18], in view of the results of this report, it may be worthwhile to focus on estimating how much effort the patient exerts consciously in each training. In our clinical setting, by adjusting the balance between torque and flexion-extension torque with a BES-based robot, the degree of effort could be set as intended in this patient with SCI. Patients and rehabilitation clinicians may have the potential to over-optimize robot settings to achieve the desired movement. However, note that the patient’s effort may not be measurable in appearance; thus, the degree of patient effort is preferably monitored using a measurement tool (e.g., VAS).

This study has some limitations. First, this case report is the result of using a BES-based robot in one case, and results are insufficient for generalization to all robot-assisted training. Second, the interval between the sessions was short, and results may have been influenced by previous sessions. Third, in other patients with different SCI severity, such as spasticity and paralysis, the relationship between patient effort and robot assist is unknown. Therefore, in the future, research will be required to search for the optimal assist condition in individuals with different degrees of SCI severity, taking into consideration the time of intervention. Fourth, this study did not compare contrasting data. No comparison was made between the HAL-SJ intervention arm and the non-intervention arm. Data on the non-intervening arm are not only contrasting, but also important in the analysis of whether motor learning has an effect on the non-intervention side. In the future, it is necessary to increase the number of cases, including a control group, and to analyze the relationship between effort and intervention effects, including non-intervention measurement. Finally, considering the negative effect of spasticity in the lower extremity on activities of daily living (ADL), these interventions may be also applicable to robot-assisted training of the lower extremity.

## 5. Conclusions

In this case, when performing upper limb training with HAL-SJ, the level of assistance with some effort reduced spasticity, whereas too strong assistance might have increased spasticity. In the HAL-SJ training for this patient with SCI, adjusting the assist settings to the patient’s effort may have resulted in better training for the spasticity and co-contraction of antagonist muscle. Further additional clinical trials are needed to determine the relationship between patient effort and optimal robot assistance.

## Figures and Tables

**Table 1:** Outcomes at the elbow before, after, and during the HAL-SJ training.

Session Number	Before HAL-SJ Training				After HAL-SJ Training				Elbow Torque with HAL-SJ at the Start of Training (Nm)			
	MAS for Biceps	MAS for Triceps	Elbow torque (Nm)		MAS for Biceps	MAS for Triceps	Elbow Torque (Nm)					
			Extension	Flexion			Extension	Flexion	Extension		Flexion	
2	2/2	1+/1+	10.3	17.4	1+/1+	1+/1+	11.2	9.9	32	(311%)	17.1	(98%)
3	2/2	2/2	9.9	15.9	1+/1+	2/2	10	11.1	24.7	(249%)	16.7	(105%)
4	2/2	2/2	10.3	16	2/1+	2/2	10.6	13.7	29	(282%)	16.6	(104%)

The torque assist setting of HAL-SJ was comfortable for the patient at the second session, less than the second session at the third session, and more than the second session at the fourth session. MAS, modified Ashworth scale; VAS, visual analog scale. MAS score is shown for the (right/left). Values (%) in parentheses indicate the rate of change in torque at the start of HAL-SJ training based on the torque immediately before training.

**Table 2:** Extent of effort before and after training without HAL-SJ and at start of training with HAL-SJ.

Extent of Effort before Training Without HAL-SJ	Extent of Effort During Training with HAL-SJ	Extent of Effort After Training Without HAL-SJ
5	1	3
9	2	2
1	0	3

The numbers indicate visual analog scale scores.

## References

[1] Lee B.B., Cripps R.A., Fitzharris M., Wing P.C. (2014). The global map for traumatic spinal cord injury epidemiology: Update 2011, global incidence rate. Spinal Cord.

[2] Katoh S., Enishi T., Sato N., Sairyo K. (2014). High incidence of acute traumatic spinal cord injury in a rural population in Japan in 2011 and 2012: An epidemiological study. Spinal Cord.

[3] National Spinal Cord Injury Statistical Center (2012). Spinal cord injury facts and figures at a glance. J. Spinal Cord Med..

[4] Dietz V., Sinkjaer T., Verhaagen J., McDonald J.W. (2012). Chapter 12–Spasticity. Handbook of Clinical Neurology.

[5] Mayo M., DeForest B.A., Castellanos M., Thomas C.K. (2017). Characterization of Involuntary contractions after spinal cord injury reveals associations between physiological and self-reported measures of spasticity. Front. Integr. Neurosci..

[6] Reinkensmeyer D.J., Patton J.L. (2009). Can robots help the learning of skilled actions?. Exerc. Sport Sci. Rev..

[7] Reinkensmeyer D.J., Aoyagi D., Emken J.L., Galvez J.A., Ichinose W., Kerdanyan G., Maneekobkunwong S., Minakata K., Nessler J.A., Weber R. (2006). Tools for understanding and optimizing robotic gait training. J. Rehabil. Res. Dev..

[8] Frullo J.M., Elinger J., Pehlivan A.U., Fitle K., Nedley K., Francisco G.E., Sergi F., O’Malley M.K. (2017). Effects of assist-as-needed upper extremity robotic therapy after incomplete spinal cord injury: A parallel-group controlled trial. Front. Neurorobot..

[9] Teramae T., Noda T., Morimoto J. (2018). EMG-based model predictive control for physical human–robot interaction: Application for assist-as-needed control. IEEE Robot Autom. Lett..

[10] Dobkin B.H. (2009). Motor rehabilitation after stroke, traumatic brain, and spinal cord injury: Common denominators within recent clinical trials. Curr. Opin. Neurol..

[11] Kirshblum S.C., Waring W., Biering-Sorensen F., Burns S.P., Johansen M., Schmidt-Read M., Donovan W., Graves D., Jha A., Jones L. (2011). Reference for the 2011 revision of the International Standards for Neurological Classification of Spinal Cord Injury. J. Spinal Cord Med..

[12] Kawamoto H., Sankai Y. (2012). Power assist method based on phase sequence and muscle force condition for HAL. Adv. Robot..

[13] Shimizu Y., Kadone H., Kubota S., Ueno T., Sankai Y., Hada Y., Yamazaki M. (2019). Voluntary elbow extension-flexion using single joint hybrid assistive limb (HAL) for patients of spastic cerebral palsy: Two cases report. Front. Neurol..

[14] The SENIAM Project (Surface ElectroMyoGraphy for the Non-Invasive Assessment of Muscles). http://www.seniam.org/.

[15] Yen J.T., Li S. (2015). Altered force perception in stroke survivors with spastic hemiplegia. J. Rehabil. Med..

[16] Marchal-Crespo L., Reinkensmeyer D.J. (2009). Review of control strategies for robotic movement training after neurologic injury. J. Neuroeng. Rehabil..

[17] Rossini P.M., Dal Forno G. (2004). Integrated technology for evaluation of brain function and neural plasticity. Phys. Med. Rehabil. Clin..

[18] Winstein C.J., Kay D.B. (2015). Translating the science into practice: Shaping rehabilitation practice to enhance recovery after brain damage. Prog. Brain Res..

